# Comparison of biochemical parameters among DPP4 inhibitor users and other oral hypoglycaemic drug users: a cross-sectional study from Anuradhapura, Sri Lanka

**DOI:** 10.1186/s41043-019-0160-x

**Published:** 2019-01-23

**Authors:** Devarajan Rathish, Channa Jayasumana, Suneth Agampodi

**Affiliations:** 1 Department of Pharmacology, Faculty of Medicine and Allied Sciences, Rajarata University of Sri Lanka, Saliyapura, Sri Lanka; 2 Department of Community Medicine, Faculty of Medicine and Allied Sciences, Rajarata University of Sri Lanka, Saliyapura, Sri Lanka

**Keywords:** Diabetes mellitus, HbA_1C_, Amylase, Lipase, AST, ALT, Pancreatitis, Hepatitis, Safety, Efficacy

## Abstract

**Background:**

Higher efficacy of incretin-based therapies for type 2 diabetes mellitus has been reported from Asia. Pancreatitis and hepatitis have also been suspected to occur due to dipeptidyl peptidase-4 inhibitor (DPP4I) treatment. The present study aims at comparing selected biochemical parameters among DPP4 inhibitor users and other oral hypoglycaemic drug users.

**Methods:**

Patients were recruited from the State Pharmaceutical Corporation, Anuradhapura, Sri Lanka, for a comparative cross-sectional study. Two groups were involved: “DPP4I” user group (*n* = 63) and “other oral hypoglycaemic” user group (*n* = 126). Mann-Whitney *U* test was performed to find a significant difference (*p* < 0.05) in the distributions of HbA_1C_, pancreatic amylase, serum lipase, AST and ALT levels between the two groups.

**Results:**

Contradicting to previous Asian studies, distribution of HbA_1C_ (*p* = 0.569) between anti-diabetic regimes with and without DPP4 inhibitors showed no significant difference. Also, amylase (*p* = 0.171), AST (*p* = 0.238) and ALT (*p* = 0.347) failed to show significance. However, lipase was significantly (*p* = 0.012) high in the DPP4I group.

**Conclusion:**

The study showed a significantly higher lipase level among the DPP4I users in comparison to other oral hypoglycaemic drug users, and possible reasons were discussed.

**Electronic supplementary material:**

The online version of this article (10.1186/s41043-019-0160-x) contains supplementary material, which is available to authorized users.

## Background

The diabetes atlas of the international diabetes federation reveals 8.8%, 8.5% and 8.6% as the prevalence of diabetes (ages 20–79) for the globe, Southeast Asia and Sri Lanka respectively [1]. An increase in type 2 diabetes mellitus (T2DM) along with the use of pesticides has been observed in Southeast Asia [2]. Organophosphate-induced disruption of glucose homoeostasis [3, 4] along with an attenuation of the “incretin effect” [5, 6] has been proposed among Asians. Furthermore, higher efficacy of incretin enhancers has been reported among Asians for T2DM [7, 8].

Along with the dysfunction of pancreatic beta cells and insulin resistance [9], patients with T2DM have shown attenuation of the incretin effect [10, 11]. There is an additional 40–60% of insulin secretion with oral glucose in comparison to the same dose of intravenous glucose; this is known as the ‘incretin effect’ [12, 13]. Glucagon-like peptide-1 (GLP-1) is one of the two most essential incretin hormones [14, 15]. These hormones increase insulin and thereby reduce blood glucose levels. Also, incretin hormones delay gastric emptying and suppress appetite. Dipeptidyl peptidase-4 (DPP4) enzyme metabolises these gut hormones [14, 15]. DPP4 inhibitors (DPP4I) are orally administered medicines which reduce the inactivation of incretin hormones and prolong their activity by inhibiting the enzyme DPP4 [16]. Thereby, they increase insulin secretion in response to meals. Sitagliptin, vildagliptin, saxagliptin, linagliptin and alogliptin belong to the DPP4I group [16–18].

Consideration of adverse effects is essential when choosing an anti-diabetic regime. Reports of acute pancreatitis (fatal and non-fatal) were available for sitagliptin, a commonly used DPP4I [16, 18]. Meta-analyses reveal no increased risk of pancreatitis with DPP4Is [19, 20]. However, most of these reviews conclude with the need for future observational studies to establish an association. T2DM itself is known to cause elevated levels of serum pancreatic-specific amylase and serum lipase [21]. Also, a pattern of increase in unknown effects may be observed when a new agent is introduced to the market, and its use becomes more widespread [22]. However, elevated serum amylase or lipase levels with DPP4Is are still a concern [23]. Also, rare risk of hepatitis has been noted with vildagliptin [18]. Immediate discontinuation is advised if the above two serious adverse effects occur [18].

The study aims to find a significant difference in efficacy using levels of HbA_1C_, among patients of Anuradhapura, Sri Lanka, who were on oral anti-diabetic regimes with and without DPP4Is; pancreatic amylase and lipase were used to compare the risk of pancreatitis; aspartate aminotransferase (AST) and alanine aminotransferase (ALT) were used to compare the risk of hepatitis. Our null hypothesis is that “there is no significant difference in the levels of HbA_1C_, pancreatic amylase, lipase, AST and ALT among T2DM patients of Anuradhapura who were on oral anti-diabetic regimes with and without DPP4Is”.

## Methods

### Study setting

This comparative cross-sectional study was conducted at the State Pharmaceutical Corporation (SPC), Anuradhapura during April–June 2017. Anuradhapura is the largest district of North-central province and in Sri Lanka by surface area. In 2012, it had a population of nearly 856,500 [24]. The majority (94.6%) belongs to the rural sector [24]. Agriculture is their primary (46%) employment [25]. Anuradhapura is also known for the use and abuse of pesticides like organophosphate [26, 27] which are implicated with T2DM [3, 4].

SPC promotes generic prescribing and sells drugs at affordable prices compared to private pharmacies [28]. The prices of 100 mg and 50 mg sitagliptin tablets at the SPC were Sri Lankan rupees 36 (USD 0.23) and 15 (USD 0.10) respectively during the study period. However, private pharmacies sold the above two at Sri Lankan rupees 74 (USD 0.48) and 46 (USD 0.30) respectively. The only outlet of SPC in Anuradhapura is situated very close to the Teaching Hospital Anuradhapura. Also, private diabetic clinics are within 500 m from the SPC, Anuradhapura. The next outlet of SPC is either in the districts of Polonnaruwa, Kurunegala or Jaffna districts which are 100, 115 and 200 km away respectively. The Teaching Hospital provides universal-free health care and is the only tertiary care hospital available for the entire North-central Province, which is maintained by the government. The above facts make Teaching Hospital, the only low-cost option for T2DM patients of Anuradhapura to seek specialised care. Although government hospitals in Anuradhapura have shown high availability of anti-diabetic agents [29], DPP4Is are not available in state-owned hospitals of Sri Lanka. Due to the above reasons, a large number of low-middle-income population visits SPC to obtain anti-diabetic drugs including DPP4Is. Oral hypoglycaemic agents were found to be within the top ten dispensed drugs at SPC [30].

### Sampling method

According to the standards of medical care in diabetes—2018 by the American diabetes association, metformin is recommended as monotherapy in type 2 diabetes mellitus unless contra-indicated [31]. Therefore, DPP4Is are rarely used as monotherapy at the local setting. Two groups were chosen for comparison. Those receiving DPP4Is (sitagliptin, linagliptin, saxagliptin, vildagliptin or alogliptin) as part of a dual or triple-drug regime were recruited to the “DPP4I group”. Those who have never received DPP4Is and were on any other dual or triple oral anti-diabetic regime were recruited to the “other oral hypoglycaemic (OOH) group”. All consecutive eligible patients presented to SPC were sampled for the OOH group until the minimum sample size was achieved. The OOH group produced a male to female ratio of 4:3. Then, patients were recruited for the DPP4I group to achieve the same male to female ratio by separately sampling all consecutive males and females who were eligible for the DPP4I group until the minimum sample size was achieved for each gender (DPP4I group male = 36; female = 27). Age and duration of diabetes mellitus were checked for a significant difference between the two groups by using the Mann-Whitney *U* test.

### Selection criteria

Inclusion criteria were the following: aged 18 to 70 years, permanent residence at Anuradhapura for ≥ 5 years, type 2 diabetes mellitus for ≤ 20 years, dual or triple oral anti-diabetic therapy for the last 3 months and not having chronic kidney disease as measured by eGFR of ≥ 60 ml/min/1.73m^2^ according to the CKD-EPI equation. Exclusion criteria were the following: any acute illness, history of parenteral anti-diabetic therapy, Morisky-Green-Levine test medical adherence score of 0–1 [32], history of chronic gastrointestinal disorders, pancreatic disorders, liver disease or malignancy, history of immunosuppression (steroid treatment or chemotherapy), everyday smoker**s** [33], heavy alcohol users [34] and pregnancy.

### Sampling size

Minimum sample sizes were calculated as 63 and 126 (1:2), for “DPP4I group” and “other oral hypoglycaemic (OOH) group” respectively using data from previous literature [35] and the formula: *n*_B_ = (1 + 1/*k*) [*σ* × (*Z*_1 − α/2_ + *Z*_1 − β_)/(*μ*_A −_ *μ*_B_)]^2^. Where, *n*_B_ is the calculated sample size for the DPP4I group (=63), *k* is *n*_A_/*n*_B_ (matching ratio) (=02), *σ* is the standard deviation (=2.3), *Z*_1 − α/2_ is the type I error (=1.96), *Z*_1 − β_ is the power (=0.8), *μ*_A_ is the OOH group mean (=7.4) and *μ*_B_ is the DPP4I group mean (=8.4).

### Instruments and investigations

Demographic data, details on co-morbidities, anthropometric measurements, blood pressure measurement and blood samples for serum creatinine, HbA_1C_, serum pancreatic-specific amylase, serum lipase, AST and ALT were obtained. Study description, obtaining written informed consent, data collection and physical examination were done by the first author in a separate room at SPC, Anuradhapura. All necessary measures were taken to preserve participant’s privacy and confidentiality.

Blood samples for the relevant investigations were analysed at the Durdans Hospital Laboratory, Anuradhapura. It is a Joint Commission International accredited hospital in Sri Lanka. Procedures for measurement of the above investigations were well established and routinely done at the above laboratory. The methods used for the analysis of serum creatinine, HbA_1C_ levels, pancreatic specific amylase, lipase, AST and ALT were enzymatic colorimetric assay, high-performance liquid chromatography, enzymatic colorimetric assay, enzymatic colorimetric assay, photometric rate (l-aspartate with 2-oxoglutarate) and photometric rate (l-alanine with 2-oxoglutarate) respectively. Quality control for HbA_1C_ was maintained using Bio-Rad lyphochek low and high control [36] and for amylase, lipase, AST and ALT using ROCHE Precinorm U and Precipath U [37].

### Data analysis and description

Data was entered to a Microsoft Excel sheet (Additional file 1). Descriptive statistics were used to describe data. Median (interquartile range) and mean (SD) were presented for the biochemical parameters focused in this study. As data were not normally distributed, Mann-Whitney *U* test was performed to determine differences between the distribution of HbA_1C_, amylase, lipase, AST, ALT, BMI, waist circumference and blood pressure of the two groups (*p* < 0.05). Additional analysis was done using the chi-square test (and Fisher exact where appropriate) to determine significant differences between the proportions having co-morbidities (*p* < 0.05).

## Results

**Fig. 1:**
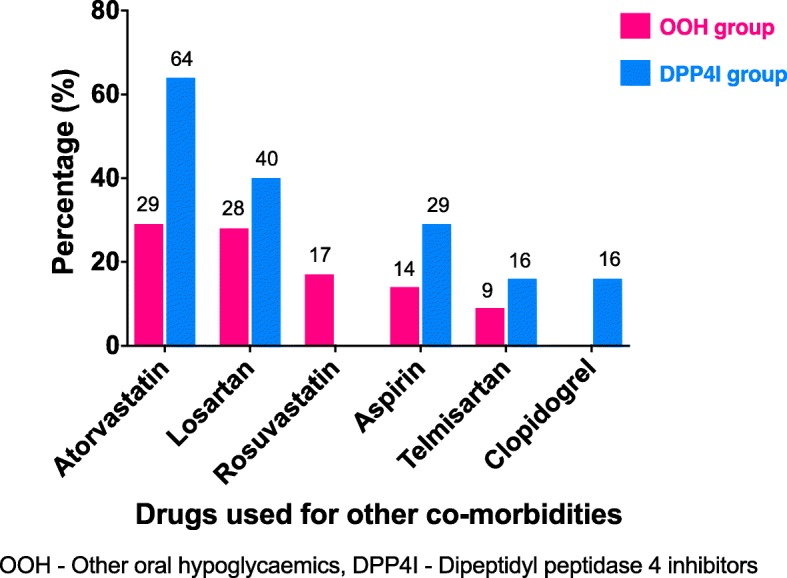
Top five drugs used by diabetic patients for other co-morbidities of the two groups, DPP4I study, Anuradhapura 2017

Most were Buddhist (OOH = 98%, DPP4I = 92%) from Nuwaragam Palata East divisional secretariat division (OOH = 43%, DPP4I = 51%) and educated up to or above the general certificate of education (advanced level) (OOH = 45%, DPP4I = 52%) among participants of both groups. Most (48%) were either unemployed or retired among patients of both the groups. The demographic data, co-morbidities, anthropometric data and blood pressure measurement for both the groups are compared in Table 1. The distribution of BMI (*p* = 0.008) and waist circumference (*p* = 0.001) for the DPP4I group were significantly different from that of the OOH group. Proportion having dyslipidaemia (*p* = 0.017) and hypertension (*p* = 0.040) was significantly high in the DPP4I group. The top five drugs used for other co-morbidities in the two groups are shown in Fig. 1.

**Table 1:** Characteristics of the study participants—DPP4I study, Anuradhapura 2017

Items	OOH group (*n* = 126)	DPP4I group (*n* = 63)	*p* value
Demography			
Median age and IQR (years)	53.5 (47–59)	54 (48–60)	0.968*
Median duration of T2DM and IQR (months)	66 (24–120)	72 (36–120)	0.204*
Co-morbidities			
Dyslipidaemia	37.3% (47/126)	55.6% (35/63)	0.017^
Hypertension	44.4% (56/126)	60.3% (38/63)	0.040^
Ischaemic heart disease	9.5% (12/126)	12.7% (8/63)	0.504^
Hypothyroidism	3.2% (4/126)	1.6% (1/63)	0.086^#^
Hyperthyroidism	1.6% (2/126)	3.2% (2/63)	0.815^#^
Asthma	1.6% (2/126)	00	0.887^#^
Osteoarthritis	0.8% (1/126)	00	0.999^#^
Anthropometric measurements and blood pressure			
Median BMI and IQR (kgm^−2^)	25.3 (23–28)	26.8 (24–30)	0.008*
Median waist circumference and IQR (cm)	92.5 (88–99)	97 (92–102)	0.001*
Median systolic blood pressure and IQR (mmHg)	138.5 (125–156)	141 (129–151)	0.749*
Median diastolic blood pressure and IQR (mmHg)	84 (76–90)	84 (79–90)	0.542*

*BMI* body mass index, *DPP4I* dipeptidyl peptidase 4 inhibitors, *IQR* interquartile range, *OOH* other oral hypoglycaemics, *T2DM* type 2 diabetes mellitus

*Mann-Whitney *U* test was performed

^^^Chi-square test was performed

^#^Fisher exact was performed

A higher median of HbA_1C_ [8.5% (69 mmol/mol)] was found among DPP4I users, compared to that of the OOH group [8.4% (68 mmol/mol)]. However, there was no significant difference in the distribution of HbA_1C_ between the two groups (*p* = 0.569). 19.1% (24/126) of OOH group had HbA1c values of < 7% (< 53 mmol/mol), whereas it was 22.2% (14/63) in the DPP4I group (chi-square statistic = 0.264, *p* = 0.608). A significant difference was observed between the distributions of serum lipase of the two groups (*p* = 0.012). A higher median of serum lipase (52 U/L) was found in the DPP4I group compared to that of the OOH group (45 U/L). However, pancreatic amylase, AST and ALT failed to show a significant difference (Table 2). Further analysis among non-dyslipidaemic patients (*n* = 107) revealed median serum lipase values of 45 U/L and 47.5 U/L for OOH (*n* = 79) and DPP4I (*n* = 28) groups respectively. The distribution of the above serum lipase levels failed to show a significant difference between the two groups (*p* = 0.246).

**Table 2:** Biochemical parameters for all types of therapies—DPP4I study, Anuradhapura 2017

Investigation	OOH group (*n* = 126)		DPP4I group (*n* = 63)		Estimated difference of means (95% CI)	*p* value*
	Median (IQR)	Mean (SD)	Median (IQR)	Mean (SD)		
eGFR (ml/min/1.73m^2^)	88.5 (73–101)	88.2 (17.0)	88 (71–98)	85.9 (16.4)	− 2.3 (− 7.4 to 2.8)	0.379
HbA_1C_ (%)	8.4 (7–10)	8.8 (1.9)	8.5 (7–10)	8.6 (1.8)	−2.0 (−0.8 to 0.4)	0.569
Pancreatic amylase (U/L)	36 (27–48)	44.8 (57.1)	38 (32–55)	47 (27.1)	2.2 (−12.8 to 17.2)	0.171
Serum lipase (U/L)	45 (35–60)	48.6 (18.6)	52 (42–73)	69.9 (67.2)	21.3 (8.6 to 34.0)	0.012
AST (U/L)	23 (17–30)	26.4 (18.4)	25 (20–32)	28.3 (15.9)	1.9 (−3.5 to 7.3)	0.238
ALT (U/L)	22 (15–33)	26 (15.5)	24 (16–34)	29.9 (19.9)	3.9 (−1.3 to 9.1)	0.347

*CI* confidence interval, *DPP4I* dipeptidyl peptidase 4 inhibitors, *IQR* interquartile range, *OOH* other oral hypoglycaemics

*Mann-Whitney *U* test was performed

Additional analysis was done for dual and triple regimes separately. 97.6% (123/126) and 11.1% (07/63) were on dual regimes for OOH and DPP4I groups respectively. HbA_1C_ for dual regimes of DPP4I users [7.5% (58 mmol/mol)] was lower in its median but was not significantly different in distribution compared to the OOH users [8.4% (68 mmol/mol)] (*p* = 0.110). Lipase for dual regimes of DPP4I users was higher in its median (60 U/L vs 45 U/L) and was significantly different in distribution compared to the OOH users (*p* = 0.007). However, pancreatic amylase, AST and ALT failed to show a significant difference (Table 3).

**Table 3:** Biochemical parameters for dual therapies—DPP4I study, Anuradhapura 2017

Investigation	OOH group (*n* = 123)		DPP4I group (*n* = 07)		Estimated difference of means (95% CI)	*p* value*
	Median (IQR)	Mean (SD)	Median (IQR)	Mean (SD)		
HbA_1C_ (%)	8.4 (7–10)	8.8 (1.9)	7.5 (7–8)	7.7 (1.2)	− 1.1 (−2.5 to 0.3)	0.110
Pancreatic amylase (U/L)	36 (27–48)	44.3 (57.6)	58 (32–97)	60.1 (33.1)	15.8 (−27.8 to 59.4)	0.095
Serum lipase (U/L)	45 (35–60)	48.7 (18.8)	60 (52–148)	91.6 (48.1)	42.9 (26.7 to 59.1)	0.007
AST (U/L)	23 (17–29)	26.2 (18.5)	23 (22–28)	24.1 (7.2)	− 2.1 (−16.0 to 11.8)	0.912
ALT (U/L)	22 (15–33)	26.1 (15.5)	24 (18–29)	23.4 (7.6)	−2.7 (−14.4 to 9.0)	0.992

*CI* confidence interval, *DPP4I* dipeptidyl peptidase 4 inhibitors, *IQR* interquartile range, *OOH* other oral hypoglycaemics

*Mann-Whitney *U* test was performed

OOH and DPP4I groups had 2.4% (3/126) and 88.9% (56/63) patients respectively on the triple regime. The overall median HbA_1C_ for the triple regime of OOH group [7.8% (62 mmol/mol)] was lower compared to that of the DPP4I group [8.5% (69 mmol/mol)]. The median of serum lipase for the triple regime of DPP4I group (51 U/L) was higher compared to that of the OOH group (45 U/L) (Table 4). As the OOH group had only 03 patients, a statistical test was not performed to find a significant difference in distribution.

**Table 4:** Biochemical parameters for triple therapies—DPP4I study, Anuradhapura 2017

Investigation	OOH group (*n* = 03)		DPP4I group (*n* = 56)		Estimated difference of means (95% CI)
	Median (IQR)	Mean (SD)	Median (IQR)	Mean (SD)	
HbA_1C_ (%)	7.8 (7–8)	7.5 (0.6)	8.5 (7–10)	8.7 (1.8)	1.2 (−0.9 to 3.3)
Pancreatic amylase (U/L)	80 (41–80)	67 (22.5)	36.5 (31–52)	45.3 (26.2)	− 21.7 (−52.6 to 9.3)
Serum lipase (U/L)	45 (41–46)	44 (2.7)	51 (40–71)	67.2 (69.1)	23.2 (−57.4 to 103.8)
AST (U/L)	27 (14–55)	32 (21)	26 (19–34)	28.9 (16.6)	− 3.1 (−23.0 to 16.8)
ALT (U/L)	16 (12–43)	23.7 (16.9)	24 (16–37)	30.8 (20.8)	7.1 (−17.4 to 31.6)

As OOH group has only 03, a statistical test was not performed to find a significant difference in distribution

*CI* confidence interval, *DPP4I* dipeptidyl peptidase 4 inhibitors, *IQR* interquartile range, *OOH* other oral hypoglycaemics

Metformin-tolbutamide combination recorded the lowest mean for HbA_1C_ [8.1% (SD 2.8)] among the dual therapies of the OOH group. All dual therapies in the DPP4I group had lower mean HbA_1C_ values than the OOH group; the lowest was seen with sitagliptin-glimepiride combination (6.2%, *n* = 01). Metformin SR-tolbutamide-pioglitazone combination recorded the lowest mean for HbA_1C_ (6.8%, *n* = 01) among the triple therapies of the OOH group; it was sitagliptin-metformin SR-glimepiride combination [(7.6% (SD 1.1)] in the DPP4I group. Sitagliptin-metformin combination recorded the highest mean for serum lipase among dual therapies of the DPP4I group. It was sitagliptin-metformin-gliclazide MR combination among triple therapies of the DPP4I group. Tables 5 and 6 summarise the mean (SD) for biochemical parameters among dual and triple therapies respectively.

**Table 5:** Comparison of means for biochemical parameters by dual therapy combinations—DPP4I study, Anuradhapura 2017

Therapy	Investigations					
	No. of patients	HbA_1C_ (%)	Pancreatic amylase (U/L)	Serum lipase (U/L)	AST (U/L)	ALT (U/L)
OOH group [mean (SD)]						
Metformin-glibenclamide	14	9.3 (1.4)	36.6 (19.2)	42.3 (21.4)	21.4 (8.2)	21.6 (15.2)
Metformin-gliclazide	36	8.3 (1.5)	39.7 (15.0)	47.8 (16.1)	23.7 (10.1)	25.2 (17.8)
Metformin SR-gliclazide	12	8.8 (1.8)	35.3 (10.5)	48 (16.1)	24.8 (8.5)	26.5 (11.7)
Metformin-gliclazide MR	12	9.3 (2.0)	53.8 (30.1)	59.4 (21.0)	29.4 (10.3)	30 (16.4)
Metformin SR-gliclazide MR	04	9.4 (2.8)	187.5 (309)	51.5 (22.2)	20.5 (3)	23.3 (5.1)
Metformin-glimepiride	31	8.9 (2.2)	38.2 (12.7)	47.6 (16.5)	24.7 (8.0)	24.9 (10.8)
Metformin SR-glimepiride	09	8.4 (2)	33.9 (11.9)	50.2 (30.6)	48.7 (56.9)	36.2 (24.1)
Metformin-tolbutamide	04	8.1 (2.8)	42 (11.3)	52 (4.2)	24.5 (3.5)	25 (7.1)
Metformin SR-tolbutamide	01	8.2	63	52	17	12
DPP4I group [mean (SD)]						
Sitagliptin-metformin	04	8 (1.4)	74.5 (36.1)	103.8 (54.1)	26.5 (6.5)	25 (7.2)
Sitagliptin-gliclazide	01	7.5	21	49	28	29
Sitagliptin-gliclazide MR	01	7.9	59	125	23	24
Sitagliptin-glimepiride	01	6.2	43	52	12	11

*DPP4I* dipeptidyl peptidase 4 inhibitors, *MR* modified release, *OOH* other oral hypoglycaemics, *SR* slow release

**Table 6:** Comparison of means for biochemical parameters by triple therapy combinations—DPP4I study, Anuradhapura 2017

Therapy	Investigation					
	No. of patients	HbA_1C_ (%)	Pancreatic amylase (U/L)	Serum lipase (U/L)	AST (U/L)	ALT (U/L)
OOH group [mean (SD)]						
Metformin-glibenclamide-pioglitazone	01	7.8	41	46	14	12
Metformin-glimepiride-pioglitazone	01	7.9	80	41	55	43
Metformin SR-tolbutamide-pioglitazone	01	6.8	80	45	27	16
DPP4I group [mean (SD)]						
Sitagliptin-metformin-gliclazide	20	9.4 (1.6)	43.4 (17.8)	58.1 (28.2)	27 (11.4)	30.7 (21.4)
Sitagliptin-metformin SR-gliclazide	06	9.1 (1.9)	36 (15.5)	55.8 (22.3)	28.7 (8.3)	29 (12.2)
Sitagliptin-metformin-gliclazide MR	05	8.6 (2)	65.6 (35.8)	96.6 (55.4)	38.2 (26.6)	50.8 (40.8)
Sitagliptin-metformin SR-gliclazide MR	02	8.4 (3.3)	44 (8.5)	67 (35.4)	20.5 (12)	32.5 (30.4)
Sitagliptin-metformin-glimepiride	10	8.1 (2)	35.7 (7.2)	41.5 (13.9)	35.9 (27.7)	25.6 (10.6)
Sitagliptin-metformin SR-glimepiride	09	7.6 (1.1)	57.8 (49.4)	109.8 (157.7)	24.9 (10.3)	29.6 (18.9)
Sitagliptin-metformin-tolbutamide	02	8.1 (2.8)	42 (11.3)	52 (4.2)	24.5 (3.5)	25 (7.1)
Vildagliptin-metformin SR-gliclazide	01	8.5	36	84	28	34
Vildagliptin-metformin SR-gliclazide MR	01	10.1	43	57	13	10

*DPP4I* dipeptidyl peptidase 4 inhibitors, *MR* modified release, *OOH* other oral hypoglycaemics, *SR* slow release

## Discussion

In contrast to previous literature [7, 8, 35, 38], this study failed to show a significantly lower HbA_1C_ with DPP4I regimes. Also, it showed a significantly higher level of lipase as against previous meta-analyses [19, 20]. T2DM patients from Anuradhapura are not benefited by DPP4Is as much as other Asians. Aetio-pathology, meal pattern, socio-cultural and pharmaco-genomic differences would have contributed. DPP4I inhibits the degradation of already secreted GLP-1. Hence, if widespread use or abuse of organophosphate [26, 27] had attenuated GLP-1 secretion [5, 6] among dwellers of Anuradhapura, DPP4Is would be less effective. However, further experiments are essential to find a definitive causality.

There were no previous similar Sri Lankan data, so data from other neighbouring Southeast Asian countries were used to compare the study findings. A Malaysian study showed a significantly low (*P* < 0.001) HbA_1C_ for DPP4I users compared to that of controls (7.4% vs 8.4%) [35]. Better glucose indices with the sitagliptin-metformin combination in comparison to the glimepiride-metformin combination were seen among South Koreans [38]. Lando et al. have shown that 36% of incretin modulator drug users had an increase in levels of serum amylase or lipase (or both) compared to 18% of the controls [23]. In Taiwan, a significantly higher risk of acute pancreatitis, within the first 2 years of the initiation of sitagliptin, was found [39]. However, two other Taiwan studies have shown no significance [40, 41]. Also, a national survey in Denmark, systematic reviews and meta-analysis have provided evidence against an increased risk of pancreatitis with the use of incretin modulators [19, 20, 42, 43].

The distributions of BMI and waist circumference in the DPP4I group were significantly higher compared to that of the OOH group. Significantly higher proportions of participants with dyslipidaemia and hypertension were seen among the DPP4I users. DPP4Is are weight neutral [16], attenuate the risk of cardiovascular disease [44] and show a significant reduction in cholesterol, low-density lipoprotein [45] and blood pressure [45]. Therefore, the present findings related to BMI, waist circumference, dyslipidaemia and hypertension might be due to the preference of DPP4Is by physicians for T2DM patients who had the above co-morbidities. However, pancreatitis secondary to dyslipidaemia is well reported [46–49]. ‘Dyslipidaemia induced pancreatitis’ most commonly present with a poorly controlled diabetes and a history of hypertriglyceridemia [47]. Another study revealed that patients with pancreatitis secondary to dyslipidaemia are predominantly obese and diabetic [48]. Therefore, dyslipidaemia could have contributed to the observed higher median of serum lipase among the DPP4I users compared to that of the OOH users. As this is a cross-sectional study, the above observation could be an example of ‘confounding by indication’. An exposure (DPP4I) looks as if associated with an outcome (pancreatitis). However, the outcome (pancreatitis) could be resulted due to an indication (dyslipidaemia) for which the exposure (DPP4I) was used [50]. Also, additional analysis among non-dyslipidaemic patients revealed no significant difference between the two groups (*p* = 0.246) in relation to the distribution of the serum lipase levels.

Glycaemic control could have been confounded by differences between the two groups in age, duration of diabetes mellitus, sex, level of adherence to treatment regime and number of anti-diabetic agents used. However, there was no significant difference in the distribution of age (*p* = 0.968) and duration of diabetes mellitus (*p* = 0.204) between the two groups. Both groups were sex matched. Only the patients with a Morisky-Green-Levine test medical adherence score of 2–4 (moderate to high) were included. Both groups had only patients who were on either dual or triple oral anti-diabetic therapy, and separate analysis on dual and triple therapies had similar findings to the overall results.

This study measured AST and ALT as these are commonly used in screening of hepatitis and are considered excellent markers of hepatocellular injury [51]. Diagnosis of hepatitis could be made using biochemical and radiological investigations. Future similar studies, using a combination of biochemical and radiological investigations to detect hepatitis, would help refine the results. This cross-sectional study cannot be expected to reveal definitive causality. However, it is unique in its findings as it was conducted in a rural agrarian district of a low-middle-income country. The study has produced an essential lead for future evaluation and monitoring.

## Conclusion

The study showed no significant difference in HbA_1C_, pancreatic amylase, AST and ALT but showed a significantly higher lipase levels among the DPP4I users in comparison to other oral hypoglycaemic drug users. A possibility of dyslipidaemia induced elevation of serum lipase was further discussed.

## Additional file

Additional file 1: DPP4Is and other oral hypoglycemic agents, Anuradhapura, Sri Lanka—2017. This contains the data of the entire study. (XLS 189 kb)

## Abbreviations

ALT: Alanine aminotransferase
AST: Aspartate aminotransferase
BMI: Body mass index
CKD-EPI: Chronic kidney disease epidemiology collaboration
DPP4I: Dipeptidyl peptidase 4 inhibitors
eGFR: Estimated glomerular filtration rate
GLP-1: Glucagon-like peptide-1
HbA_1C_: Glycated haemoglobin
MR: Modified release
OOH: Other oral hypoglycaemics
OPI: Organophosphorus insecticides
SPC: State Pharmaceutical Corporation
SR: Slow release
T2DM: Type 2 diabetes mellitus
USD: United States Dollars
